# Beyond Synthetics: Promising Outcomes With the Invengenx® Bovine Pericardial Patch for Ventricular Septal Defect Repair in a Young Pediatric Population

**DOI:** 10.7759/cureus.55530

**Published:** 2024-03-04

**Authors:** Vishal V Bhende, Tanishq S Sharma, Mathangi Krishnakumar, Anikode S Ramaswamy, Kanchan Bilgi, Sohilkhan R Pathan

**Affiliations:** 1 Pediatric Cardiac Surgery, Bhanubhai and Madhuben Patel Cardiac Centre, Shree Krishna Hospital, Bhaikaka University, Karamsad, IND; 2 Anesthesiology, Saint John's Medical College Hospital, Bengaluru, IND; 3 Pathology, People's Education Society (PES) Institute of Medical Sciences and Research, Kuppam, IND; 4 Neuroanesthesiology, People Tree Hospitals, Bengaluru, IND; 5 Clinical Research Services (CRS), Bhanubhai and Madhuben Patel Cardiac Centre, Shree Krishna Hospital, Bhaikaka University, Karamsad, IND

**Keywords:** congenital heart disease (chd), ventricular septal defect closure, pediatric cardiac surgery, invengenx® bovine pericardial patch, ventricular septal defects

## Abstract

Ventricular septal defects (VSDs) are a prevalent congenital heart anomaly demanding safe and lasting interventions. This paper explores the application of Invengenx® bovine pericardial patch (Tisgenx, Irvine, California), a promising biomaterial, in VSD repair. We present two case studies: a seven-month-old infant and a three-year-old child undergoing VSD closure using autologous and bovine pericardial patches, respectively.

Both patients tolerated the procedures well, experiencing no intra-operative complications and demonstrating excellent postoperative recovery. Echocardiography postoperatively showed no complications and improved clinical outcomes. Notably, the pericardial patches exhibited excellent integration and suture retention, highlighting their durability and compatibility with the growing heart.

These cases establish the feasibility and effectiveness of the Invengenx® pericardial patch for VSD repair. The favorable outcomes in terms of safety and efficacy support the potential of this biomaterial as a valuable alternative in pediatric cardiac surgery, particularly for complex VSDs or patients with contraindications to synthetic patches. Further research is crucial to unlock the full potential of bovine pericardium as a durable and advantageous option for VSD repair in a broader range of pediatric patients.

## Introduction

Ventricular septal defects (VSDs) are a prevalent form of congenital heart disease, affecting roughly 37% of cases. Treatment relies heavily on the defect characteristics, with large VSDs demanding surgical intervention when pressures in the right ventricle (RV) and pulmonary artery (PA) approach those in the left ventricle and aorta [1]. While traditional repair methods have proven successful, the quest for novel biomaterials in this field continues.

A wide range of biomaterials, from synthetic to biological and even patient-derived (autologous) options, have been explored for correcting congenital heart defects in children and adults [2-5]. Among these, xenogeneic (animal-derived) extracellular matrix biomaterials, like bovine pericardium, hold a prominent position. Since their pioneering use in prosthetic heart valves, bovine pericardium has become a mainstay in cardiac surgery, demonstrating efficacy in repairing atrial septal defects (ASDs) and VSDs, and reconstructing the right ventricular outflow tract (RVOT) [6-7].

However, glutaraldehyde, the common fixative for bovine pericardium, raises concerns. Its toxicity can trigger immune responses and contribute to implant failure over time, primarily through rigidity, calcification, and shrinkage [8,9]. Although complications in ASD and VSD closure using bovine pericardium are rare, potential issues include thrombus formation, aneurysm development, and patch dehiscence [10-12]. While glutaraldehyde-treated bovine pericardium has a lengthy track record in VSD closure, debate persists regarding its long-term success rate and potential side effects like minor redirection of blood flow, cardiac conduction delay, and blocks, inadequate aortic valve closure, stiffening of the patch, and calcification [10-13].

This highlights the need for innovative biomaterials that address these limitations. The Invengenx® bovine tissue patch (Tisgenx, Irvine, California) features the proprietary elixPTM™ technology. This preserves the intricate triple-stranded helical arrangement of collagen molecules, both within and between fibers. This translates to reduced antigenicity, preserving the tissue's original formation while enhancing its mechanical strength. Importantly, elixPTM™ maintains the patch's flexibility, minimizing unwanted reactions like suture line bleeding, delamination, and inflammation [14]. We report two cases of VSD repair, one with the Invengenx® bovine pericardial tissue patch and one with an autologous pericardial patch.

## Case presentation

The Institutional Ethics Committee (IEC-2) at H.M. Patel Centre for Medical Care and Education, Anand, Gujarat (Approval No. IEC/BU/2024/Cr.09/42/2024, dated 12.02.2024) approved this study. The authors assert that there is no identifiable personal information in this report regarding the patients. Given that the patients are minors, informed written consent was obtained from their parents.

We report two cases of VSD repair, one with the Invengenx® bovine pericardial tissue patch and one with an autologous pericardial patch. Functional status assessments before and after surgery employed the New York Heart Association (NYHA) classification and the modified Ross score.

Case one (P_1_)

The patient was a seven-month-old female diagnosed with double outlet right ventricle (DORV), ventricular septal defect (VSD), and bilateral superior vena cava (SVC), accompanied by severe pulmonary arterial hypertension (PAH). She weighed 7.6 kilograms. Her cardiac function was classified as class I according to the New York Heart Association (NYHA) functional classification, and her condition was scored as one on the Modified Ross Score, indicating minimal heart failure symptoms at the time of evaluation.

Intra-operative Technique

Median sternotomy: The surgeon performed a median sternotomy, creating a vertical incision down the center of the chest to access the underlying pericardium and heart chambers.

Cardiopulmonary bypass (CPB) establishment: To maintain blood flow throughout the body during the surgery, cardiopulmonary bypass was established. This involved cannulating the aorta, superior vena cava (SVC), and inferior vena cava (IVC), diverting blood through an external circuit that oxygenated and pumped it back to the body. Efficient myocardial protection was prioritized through a modern, multi-faceted approach. This involved utilizing a hypothermic cardiopulmonary bypass circuit set at 28°C, supplemented with antegrade cardioplegia with multi-dose Del-nido crystalloid, topical cooling, and concluding with antegrade reperfusion with warm blood.

Right atrial (RA) approach: Precise openings were created in the right atrium to expose the VSD from within the heart chambers.

**Figure 1:**
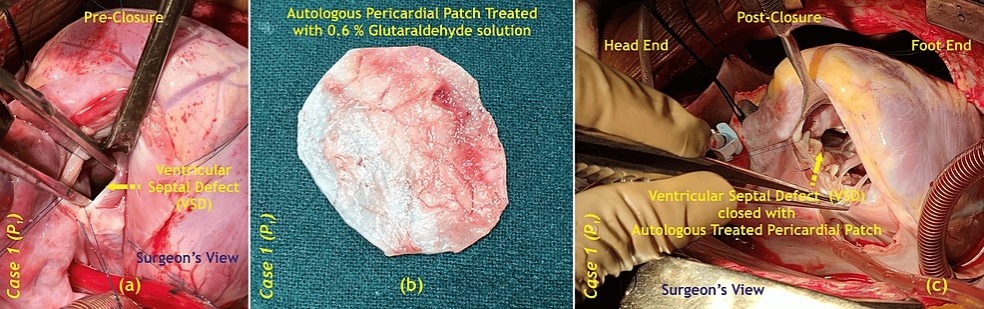
Intraoperative VSD closure with an autologous pericardial patch of case one (P1); (a) pre-closure, (b) patch preparation, (c) post-closure VSD - ventricular septal defect Image credits: Dr. Vishal V. Bhende

Precise patch design: An autologous pericardial patch, meticulously sized and treated with glutaraldehyde to enhance durability and minimize immune response, was prepared for closure of the VSD (Figure 1).

Watertight closure: The patch was carefully positioned over the VSD and secured with continuous sutures, ensuring total closure of the defect and preventing any residual shunting of blood between the heart chambers.

CPB weaning and hemodynamic confirmation: Following careful assessment of cardiac function and bleeding control, the patients were gradually weaned off cardiopulmonary bypass, allowing the heart to resume its natural rhythm. The surgical procedure involved a cross-clamp time of 93 minutes and a cardio-pulmonary bypass duration of 175 minutes.

Closure of RA incisions and sternotomy: The openings in the right atrium were meticulously closed with sutures, followed by approximation and secure closure of the sternum.

Postoperatively, the patient was shifted to cardiac surgical intensive care unit (CSICU). The patient's extubation was successfully performed after 25 hours. The patient's stay in CSICU lasted for five days, leading to a total hospital stay of nine days. The patient experienced no complications such as infection, bleeding, the need for re-exploration, low cardiac output, atrioventricular (AV) complete block, mediastinitis, or pulmonary hypertension (PHT) crisis during this period. The pulmonary-to-systemic blood flow ratio (Qp/Qs) was measured at 2:1, indicating a balanced circulatory flow. There were no residual defects observed, and no re-exploration was required.

**Figure 2:**
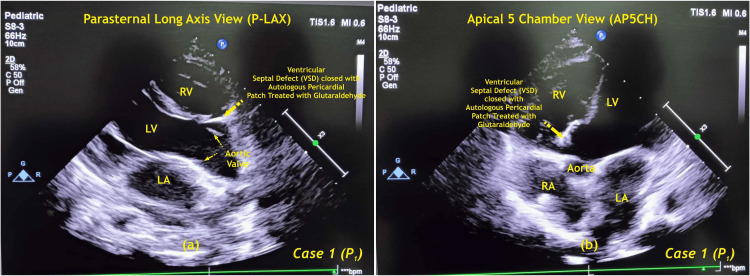
Postoperative 2D echocardiography of case one (P1) RA - right atrium; LA - left atrium; RV - right ventricle; LV - left ventricle; P - patient Image Credits: Dr. Vishal V. Bhende

Transthoracic echocardiography (ECHO) confirmed a successful closure of the VSD and assessed the function of the heart chambers and valves. ECHO was done at discharge from the hospital. Complications related to the patch, like aneurysm formation, dehiscence, calcification, thrombus, or vegetation, were not seen (Figure 2). 

The pre- and postoperative ECHO findings were compared, showing significant changes (Table 1).

**Table 1:** Comparison between preoperative and postoperative ECHO findings of case one (P1) VSD - ventricular septal defect; TAPSE - tricuspid annular plane systolic excursion; EF - ejection fraction

Feature	Case one preoperative	Case one postoperative
Ventricular septal defect (VSD)	Large sub-aortic VSD with significant over-ride, shunting left to right, divided into two with inter-ventricular septum aneurysm	Small residual VSD (45 mm Hg peak gradient), shunting left to right
Atrial septal defect (ASD)	N/A	N/A
Pulmonary valve regurgitation	Mild	Mild
Mitral valve regurgitation	N/A	Mild
Tricuspid valve regurgitation	N/A	N/A
Pulmonary arterial hypertension (PAH)	Severe	Mild
Right ventricle function	Dilated	N/A
Left ventricle function	N/A	N/A
Other findings	Inter-ventricular septum aneurysm	VSD patch in-situ

Case two (P_2_)

A three-year-old male was diagnosed with a ventricular septal defect (VSD), mild tricuspid valve regurgitation, mild mitral valve regurgitation, and severe pulmonary arterial hypertension (PAH). At the time of evaluation, he weighed 10.5 kilograms. His cardiac function was classified as class I according to the NYHA functional classification, indicating no limitation of physical activity. His condition was also scored as 1 on the modified Ross score, reflecting minimal heart failure symptoms.

**Figure 3:**
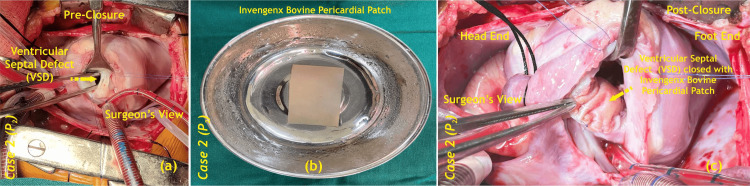
Intraoperative VSD closure with a bovine pericardium (Invengenx®) patch; (a) pre-closure, (b) patch preparation, (c) post-closure VSD - ventricular septal defects; P - patient Image credits: Dr. Vishal V. Bhende

The patient underwent VSD closure with a bovine pericardium (Invengenx®) patch, the intraoperative technique is similar to that described in case one. In this patient, during the intra-operative phase, the cross-clamp time was recorded at 50 minutes, and the cardio-pulmonary bypass (CPB) duration was 76 minutes (Figure 3).

**Figure 4:**
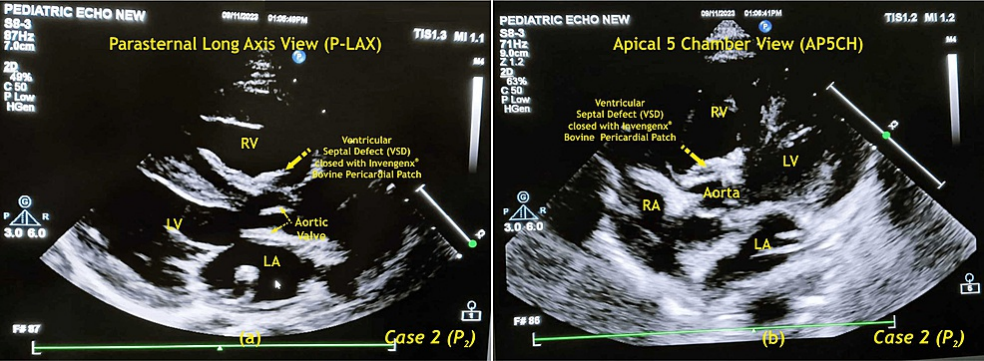
Postoperative 2D echocardiography of case two (P2) RA - right atrium; LA - left atrium; RV - right ventricle; LV - left ventricle; P - patient Image credits: Dr. Vishal V. Bhende

Postoperatively, the patient remained stable and was shifted to CSICU. The process of extubation was carried out successfully at 96 hours. The patient's subsequent stay in the CSICU spanned eight days, leading to an overall hospitalization duration of 13 days. Throughout the postoperative phase, the patient did not exhibit any complications such as infection, bleeding, requirement for re-exploration, low cardiac output, AV complete block, mediastinitis, or a pulmonary hypertension (PHT) crisis. The ratio of pulmonary-to-systemic blood flow (Qp/Qs) was recorded at 2:1. Despite the presence of a residual defect measuring 2.2 mm, there was no indication for re-exploration during the patient's recovery period. An echocardiography was done in the postoperative period, which showed an intact patch (Figure 4).

The postoperative echocardiography was compared to the preoperative findings (Table 2). 

**Table 2:** Comparison between preoperative and postoperative ECHO findings of case two VSD - ventricular septal defect; TAPSE - tricuspid annular plane systolic excursion; EF - ejection fraction

Feature	Case two preoperative	Case two postoperative
Ventricular septal defect (VSD)	Large sub-aortic VSD, extending posteriorly, malaligned septum, bidirectional shunting (dominant left to right)	Residual VSD (34 mm Hg peak gradient), shunting left to right
Atrial septal defect (ASD)	Small ostium secundum ASD, shunting left to right	None
Pulmonary valve regurgitation	Mild	N/A
Mitral valve regurgitation	Mild	Mild
Tricuspid valve regurgitation	Mild	Severe with septal leaflet restriction (50 mm Hg peak gradient)
Pulmonary arterial hypertension (PAH)	Severe	N/A
Right ventricle function	N/A	Mild dysfunction (TAPSE: 10.6 mm, EF: 37%)
Left ventricle function	N/A	Mild dysfunction (EF: 50%)
Other findings	N/A	N/A

Results

Overall, both patients had a significant recovery with VSD repair. Both patients had a large left to right shunt in the preoperative period, with improvement after surgery. There were no reported patch-related complications; the peri-operative period was uneventful. With progressive physical therapy and rehabilitation, both patients had good functional recovery with NYHA I.

## Discussion

Ventricular septal defect (VSD) repair remains a cornerstone of pediatric cardiac surgery, with recent studies reporting encouraging outcomes and low complication rates. Early intervention is crucial to prevent pulmonary hypertension and improve long-term prognosis. Successful closure can reduce endocarditis risk, mitigate pulmonary hypertension, and improve overall survival [15]. Bovine pericardial tissue has gained favor in reconstructive heart surgery due to its advantageous properties, particularly during procedures involving a small aortic annulus, aortic root abscess, or post-infarction VSD. We favor its use for its excellent handling characteristics, inherent elasticity, and reduced risk of infective endocarditis compared to alternative materials [2]. While some studies have documented potential downsides of synthetic and autologous pericardial patches, including aneurysmal changes, calcification, and shrinkage, biological options have dominated the past 25 years. Their superior biocompatibility, minimal infectious risk, and reduced thrombogenicity are compelling advantages [1,2].

The pioneering use of glutaraldehyde-preserved bovine pericardial patches in cardiac surgery dates back to 1977, when it was employed by Ionescu et al. within prosthetic valves [6]. Its ease of manipulation, robust strength, and resistance to shrinkage during intracardiac implantation solidified its potential as an ideal patch material for various intra-cardiac repairs. This success story led to its widespread adoption in contemporary cardiovascular surgery for diverse applications [16]. The surgeon's preference determines the patch material. Synthetic options like Dacron or Gore-Tex are common, with Dacron's ability to induce fibrosis sometimes aiding the closure of small residual leaks in the early postoperative period. Both the patient's own tissue and pericardium sourced from bovine or equine can also serve as options. Nonetheless, fresh, untreated pericardium poses handling challenges and risks long-term shrinkage and stretching [17].

A study by Joseph et al. provides compelling evidence supporting the use of autologous pericardium treated with glutaraldehyde for VSD closure. Compared to Gore-Tex, this material demonstrated significantly lower complication rates, superior handling characteristics, improved compatibility with septal movements, and a reduced incidence of postoperative infective endocarditis. Given these advantages, particularly its cost-effectiveness, glutaraldehyde-treated pericardium presents a valuable alternative for VSD repair in resource-limited settings [18] Bhende et al. in their study of utilizing bovine pericardium (Invengenx®) patch for patients with tetralogy of Fallot demonstrated good handling characteristics and complication-free postoperative and follow up period [14].

This report showcases successful VSD closure with a bovine pericardium (Invengenx®) patch using a meticulous surgical technique and established postoperative management protocols. The utilization of bovine pericardial patches provides a durable and reliable solution, demonstrating excellent outcomes. However, additional research with a wider patient population and an extended follow-up period is required to ascertain the findings of this report.

## Conclusions

Surgical closure of VSDs using Invengenx® bovine pericardial patches remains an effective and established intervention, restoring normal hemodynamics and improving prognosis. Careful attention to surgical technique and meticulous postoperative management contribute to successful outcomes and enhanced quality of life for patients.
